# Activities of daily living questionnaire from patients’ perspectives in Parkinson’s disease: a cross-sectional study

**DOI:** 10.1186/s12883-016-0600-9

**Published:** 2016-05-21

**Authors:** Su-Yun Lee, Sung Kwan Kim, Sang-Myung Cheon, Jung-Wook Seo, Min Ah Kim, Jae Woo Kim

**Affiliations:** Department of Neurology, Dong-A University School of Medicine, 3-1, Dongdaesin-dong, Seo-gu, Busan, Korea 607-020; Department of Prevention and Management of Regional Cardiocerebrovascular Center, Dong-A University Medical Centre, Busan, Korea; Department of Physical Medicine and Rehabilitation, Changwon Hanseo Rehabilitation Hospital, Changwon, Korea

**Keywords:** Parkinson’s disease, Activities of daily living, Questionnaire

## Abstract

**Background:**

The aim of this study was to develop an assessment tool for activities of daily living (ADL) from the perspective of patients with Parkinson’s disease (PD) and examine the validity and reliability of the assessment.

**Methods:**

A preliminary 45-item questionnaire was developed through intensive interviews with 54 patients with PD and administered to another group of 248 patients with PD. Based on clinical and statistical analyses, 20 ADL-items were selected. The final 20-item questionnaire was examined in the other group of 59 patients with PD.

**Results:**

The new ADL questionnaire showed high internal consistency (Cronbach’s α, 0.962–0.966) and acceptable test-retest reliability (0.632–0.984). Concurrent validity was shown as a significant positive correlation between the new ADL questionnaire and other ADL or clinical instruments. The Hoehn and Yahr stage showed the highest degree of correlation with the new ADL questionnaire, followed by the other ADL scales (Schwab and England ADL and the ADL subscore of the Unified Parkinson’s Disease Rating Scale). Additionally, a regression analysis was conducted with the disease-specific quality of life questionnaire, and the new ADL questionnaire was the most powerful predictor of quality of life among the clinical instruments.

**Conclusions:**

The new ADL questionnaire is a valid tool for assessing ADL from the perspectives of patients with PD.

## Background

Parkinson’s disease (PD) is a chronic disorder due to the progressive degeneration of dopamine-producing cells in brain structures, including the substantia nigra [1]. PD is characterized by motor disturbances, such as tremor, bradykinesia, rigidity, and postural instability. Clinical presentations of PD also include a variety of non-motor symptoms. Thus, patients with PD are vulnerable to decreased activities of daily living (ADL), such as walking, talking, swallowing, or simple tasks like bathing or dressing [2].

Despite the recent development of medical, surgical, and rehabilitative treatments, PD remains a progressive condition; there are no proven management strategies to reverse or halt its pathological processes. Patients with PD are at increased risk for decreased activities of daily living (ADL) with progression of the disease, particularly those requiring coordination and balance [3–5]. The treatment goal for patients with PD is to focus on symptoms and improve ADL. Therefore, it is mandatory to assess the ADL of patients with PD, which is essential for evaluating the effectiveness of symptomatic and potentially disease-modifying treatments.

Some studies have focused on the ADL of patients with PD. However, these studies have revealed limitations due to a lack of reliable and valid tools for assessing ADL from the patient’s perspective [6–10]. The Unified Parkinson’s Disease Rating Scale (UPDRS) is widely used to assess mentation, ADL, motor symptoms, and treatment complications in patients with PD [11]. The advantages of the ADL subsection of the UPDRS have been well described [12, 13]. However, it has been criticized due to its ambiguity and redundancy of items, such as items overlapping in the ADL and motor sections, and a lack of the patient’s perspective has also been reported [11, 14]. It also requires an interview by an examiner. The new version of the UPDRS includes motor experiences associated with ADL that are self-evaluated by patients or their caregivers [14]. However, the new version does not differ greatly from the old version and fails to evaluate ADL comprehensively.

Improvements in motor symptoms, which are unresponsive to medical and surgical treatments, have been observed in patients with PD with recent advancements in rehabilitation therapy [15]. These symptoms are related to balance, walking, and mobility, all of which constitute the ADL of patients with PD. However, the UPDRS shows limited validity for assessing the effects of physiotherapy on balance and mobility, both of which are essential when measuring the ADL of patients with PD [16].

We developed a new ADL questionnaire from the patient’s perspective and assessed its validity and reliability in this cross-sectional study.

## Methods

### Study patients

Patients with PD were recruited consecutively from an outpatient clinic in a specialized center of a referral hospital. Patients were diagnosed with clinically probable PD according to the United Kingdom Parkinson’s Disease Society Brain Bank clinical diagnostic criteria [17]. Patients with cognitive impairment based on a Korean Mini-Mental Status Examination (K-MMSE) score < 24 and patients without sufficient cognitive or physical ability to cooperate were excluded [18]. Patients who had other medical problems that seriously affected ADL, such as stroke, osteoarthritis, and angina, were also excluded.

### Development of the preliminary 45-item questionnaire

Movement disorder and neurorehabilitation specialists reviewed motor aspects of disabilities of daily living in patients with PD with reference to various ADL scales and collected ADL items [19–25]. An open and intensive interview was performed with 54 patients with PD; they were asked whether they had disability in a collection of ADL items and were asked to add any other ADL items experienced during their daily lives. The preliminary 45-item ADL questionnaire was prepared and included household, outdoor, and social activities (Appendix 1).

### Selection of the final 20-item questionnaire

Another group of 248 patients with PD (90 men and 158 women; mean age, 67.9 ± 9.2 years; mean K-MMSE score, 26.2 ± 3.8 points; mean Hoehn and Yahr [HY] stage, 2.4 ± 0.6) were asked to select the items that they found difficult in their daily living and were asked to select the three items that were most important to them using the preliminary 45-item questionnaire.

Clinical and statistical analyses were performed to select the ADL items. The frequency of items reflecting patient’s experience and importance and clinical significance for infrequent items, such as swallowing, were included in the clinical analyses. Statistical analyses were conducted using maximum likelihood to estimate the factor structure matrix. The number of items was determined using parallel analysis and the scree test. We used the Promax (Kappa = 2) oblique rotation method to simplify the structure, and each item was selected and classified based on a factor loading > 0.4.

All items were given a score on a 5-point scale (0: no impairment of ADL and 5: inability to perform ADL) (Appendix 2).

### Validation of the final 20-item questionnaire

Fifty-nine patients with mild-to-moderate PD were additionally recruited to validate the final 20-item questionnaire (Table 1). We assessed internal consistency of the new ADL questionnaire using Cronbach’s α. In addition, we performed repeated interviews at a 4-week interval to assess test-retest reliability. Concurrent validity was analyzed by the correlation between the new ADL questionnaire and other clinical instruments including the UPDRS, the Schwab and England ADL (SEADL) scale, Beck’s Depression Inventory [26], the Autonomic Dysfunction Questionnaire [27], the Fatigue Severity Scale [28], and the Parkinson’s disease quality of life (PDQL) questionnaire [29]. A simple regression analysis was conducted for the PDQL questionnaire to evaluate the influence of various clinical instruments on quality of life.

**Table 1:** Baseline and clinical characteristics of the 59 patients who were evaluated to validate the 20-item questionnaire

	Mean ± SD
Number (male-to-female ratio)	59 (22:37)
Age (years)	66.85 ± 8.25
Disease duration (years)	6.14 ± 4.80
Treatment duration (years)	4.43 ± 4.00
The daily dose of levodopa (mg/day)	540.00 ± 296.21
K-MMSE	26.49 ± 2.85
New questionnaire	23.24 ± 22.29
HY stage	2.42 ± 0.58
UPDRS	45.78 ± 18.49
Mentation	3.26 ± 2.05
ADL	11.88 ± 6.22
Motor	27.91 ± 11.05
Complications	2.73 ± 2.97
SEADL	77.59 ± 15.89
BDI	22.38 ± 11.53
PDQL	115.53 ± 39.93
Parkinson’s symptoms	42.51 ± 14.14
Systemic symptoms	25.27 ± 9.64
Social function	19.21 ± 7.18
Emotional function subscore	28.54 ± 10.80
ADQ	19.21 ± 12.42
FSS	36.20 ± 15.69

*K-MMSE* Korean Mini-Mental Status Examination, *HY* Hoehn and Yahr, *UPDRS* Unified Parkinson’s Disease Rating Scale, *ADL* Activities of Daily Living, *SEADL* Schwab and England ADL, *BDI* Beck’s Depression Inventory, *PDQL* Parkinson’s Disease Quality of Life, *ADQ* Autonomic Dysfunction Questionnaire, *FSS* Fatigue Severity Scale

### Statistical analysis

All data are expressed as mean ± standard deviation for continuous variables and as proportions of patients (%) for categorical variable. The univariate analysis included Student’s *t*-test and Pearson’s chi-square test, as appropriate, to analyze differences between the two groups. A correlation analysis was conducted using Spearman’s method. The statistical analysis was performed using SPSS ver. 19.0 for Windows software (SPSS Inc., Chicago, IL, USA). A *P*-value ≤ 0.05 was considered significant.

## Results

### Evaluation of ADL with the preliminary 45-item questionnaire in 248 patients with PD

The most common ADL items experienced by patients were getting up (61.7 %), dressing (57.7 %), and moving objects (56.9 %) (Table 2). The most important ADL items selected by patients were “walking outside”, “using a spoon and chopsticks”, and “getting up”. Some differences in the frequencies for selected items were detected according to sex, such as “driving” for men, “preparing a meal”, “washing dishes”, “cleaning”, and “washing clothes” for women (chi-square test, *P* ≤ 0.05).

**Table 2:** The most common disabilities during daily activities in patients with Parkinson’s disease using the preliminary 45-item questionnaire

Items of ADL	Number	Percent
Getting up from the floor	153	61.7
Dressing	143	57.7
Moving objects	141	56.9
Wearing shoes	136	54.8
Writing	134	54.0
Walking inside	133	53.6
Walking outside	131	52.8
Standing	130	52.4
Turning around in bed	126	50.8
Sitting on the floor	126	50.8

*ADL* Activities of Daily Living

A positive correlation was observed between the number of items patients found difficult and severity of the disease, as shown by the HY stage (Table 3).

**Table 3:** The most common and important items in patients with Parkinson's disease according to Hoehn and Yahr stage using the preliminary 45 item questionnaire

HY stage	Number of items	Most common items	Most important items
1	1.29 ± 1.60	Getting up from the floor	Exercise
		Sitting on the floor	Walking outside
		Walking outside	Grasping and releasing objects
2	11.31 ± 11.06	Dressing	Walking outside
		Getting up from the floor	Getting up from the floor
		Writing	Using a spoon and chopsticks
2.5	20.30 ± 12.04	Getting up from the floor	Walking outside
		Transferring objects	Sitting on the floor
		Wearing shoes	Getting up from the floor
3	24.18 ± 12.81	Getting up from the floor	Walking outside
		Moving objects	Taking the first step
		Taking the first step	Getting in/out of bed
4–5	34.50 ± 7.52	Dressing	Waking outside
		Getting in/out of a car	Using the toilet
		Taking a bath/shower	Getting in/out of bed

*HY* Hoehn and Yahr

### Validation of the new ADL questionnaire in 59 patients with PD

The internal consistency of the new questionnaire was acceptable with an item-total correlation (range, 0.523–0.898) and high consistency, as shown by Cronbach’s α of 0.962–0.966. Test-retest reliability was acceptable, reached at 0.632–0.984. Concurrent validity was detected as significant correlations between scores on the new ADL questionnaire and other ADL scales (SEADL and the ADL subscore of the UPDRS). The other clinical instruments were also correlated with the new ADL questionnaire, except the MMSE (Table 4).

**Table 4:** Correlations between the new questionnaire and other clinical instruments

	Coefficient	*P-*value
K-MMSE	−0.066	0.621
HY stage	0.682	<0.001
SEADL	−0.617	<0.001
UPDRS	0.521	<0.001
Mentation’	0.313	0.021
ADL’	0.608	<0.001
Motor’	0.322	0.018
Complications’	0.562	<0.001
BDI	0.586	<0.001
PDQL	−0.605	<0.001
Parkinson’s symptoms’	−0.596	<0.001
Systemic symptoms’	−0.527	<0.001
Social function’	−0.589	<0.001
Emotional function’	−0.594	<0.001
ADQ	0.480	0.000
FSS	0.449	0.001

*K-MMSE* Korean Mini-Mental Status Examination, *HY* Hoehn and Yahr, *SEADL* Schwab and England ADL, *UPDRS* Unified Parkinson’s Disease Rating Scale, *ADL* Activities of Daily Living, *BDI* Beck’s Depression Inventory, *PDQL* Parkinson’s Disease Quality of Life, *ADQ* Autonomic Dysfunction Questionnaire, *FSS* Fatigue Severity Scale

The regression analysis for quality of life showed that the new ADL questionnaire was the most powerful predictor for quality of life in patients with PD followed by the ADL subscore of the UPDRS (Table 5). The new questionnaire and the ADL subscore of the UPDRS were the strongest predictors of the PDQL after adjusting for age, disease duration, treatment, and daily levodopa dosage (adjusted *R*^*2*^ values, 0.456 and 0.464, respectively).

**Table 5:** Results of a simple regression analysis for the Parkinson’s Disease Quality of Life Score

	*P-*value	*R* ^*2*^
New questionnaire	<0.001	0.366
K-MMSE	0.746	0.002
HY stage	0.003	0.162
SEADL	<0.001	0.280
UPDRS	<0.001	0.232
Mentation’	0.005	0.150
ADL’	<0.001	0.335
Motor’	0.035	0.088
Complications’	<0.001	0.247
BDI	0.002	0.181
ADQ	0.011	0.128
FSS	0.238	0.030

*K-MMSE* Korean Mini-Mental Status Examination, *HY* Hoehn and Yahr, *SEADL* Schwab and England ADL, *UPDRS* Unified Parkinson’s Disease Rating Scale, *ADL* Activities of Daily Living, *BDI* Beck’s Depression Inventory, *ADQ* Autonomic Dysfunction Questionnaire, *FSS* Fatigue Severity Scale

## Discussion

The number of ADL items that were difficult for patients with PD increased according to the HY stage using the 45-item questionnaire, indicating that progression of the disease increased ADL impairment. This finding suggests that items on the preliminary questionnaire covered ADL throughout the disease stages and comprehensively reflected the patient’s perspectives.

Some differences were found in the list of selected items according to HY stage. However, the most common and important items for the patients were similar at all HY stages. Getting up was the most common, and walking outside was the most important activity. These findings suggest that mobility deserves special attention compared to others activities during the daily lives of these patients.

Among the most frequently selected 10 items, only four (dressing, writing, walking, and turning around in bed) are present in the ADL section of the UPDRS. These findings suggest that the ADL section of the UPDRS may be insufficient to reflect the daily disabilities of patients with PD.

The validity of the new questionnaire was acceptable, as shown by the correlation with other clinical measures. Concurrent validity was reasonable based on the high correlation of the new questionnaire with other ADL and clinical instruments, except the K-MMSE. Given that cognitive function can greatly affect ADL, this result may be distorted due to the limited distribution of the K-MMSE scores based on the exclusion criteria of this study. The new questionnaire showed the highest degree of correlation with HY stage and also had a high degree of correlation with other clinical instruments, such as the SEADL and the ADL section of the UPDRS. Moreover, the motor subscore of the UPDRS was marginally correlated with the new ADL questionnaire, but it was less correlated than depression, autonomic dysfunction, and fatigue. This finding is consistent with a recent report that severity of motor symptoms, as expressed by the motor subscore of the UPDRS, has less impact on the health status of patients with PD than non-motor symptoms [30].

Quality of life may be the most important clinical measure representing the current status of patients with PD. Our results show that the new questionnaire was a strong predictor of quality of life in patients with PD among the clinical instruments evaluated in this study. A regression analysis showed that the new questionnaire and the ADL subscore of the UPDRS had strong predictive value for quality of life in patients with PD after adjusting of for age, disease duration, treatment, and daily levodopa dosage.

Several limitations of this study should be discussed. Different importance could be assigned to some items in different cultures, such as “getting up from or sitting on the floor”. Thus, it may be necessary to validate the new questionnaire in different cultural areas. Also, there could be some biases from the predominance women in recruited patients at the evaluation of preliminary 45-item questionnaire. And, the new questionnaire showed relatively weak correlation with motor part of UDPRS, compared to other scales. This finding could suggest that the new questionnaire might not be sensitive enough as an assessment tool for motor changes responsive to physical therapy. Finally, this study was conducted using a cross-sectional design. Therefore, we failed to assess the feasibility of the new questionnaire for detecting changes along the course of the disease. This deserves further longitudinal studies.

Despite these limitations, the new questionnaire has several advantages over other clinical instruments; it reflects actual patient disabilities during daily living based on a patient’s perspective. Changes in the ADL of patients could be easily and effectively evaluated, because the questionnaire is designed for self-administration. Thus, the new questionnaire is applicable to long-term follow-up in an actual clinical setting.

## Conclusion

The new questionnaire developed based on the perspective of patients with PD was found to be a valid tool for assessing ADLs of patients with PD.

### Ethics and consent to participate

This study was approved by the Institutional Review Board of Dong-A University Hospital (No. 14–101) and patients submitted written informed consent.

### Consent to publish

Not applicable

### Availability of data and materials

All the data supporting our findings is contained within the manuscript.

## Figures and Tables

**Table 7:**
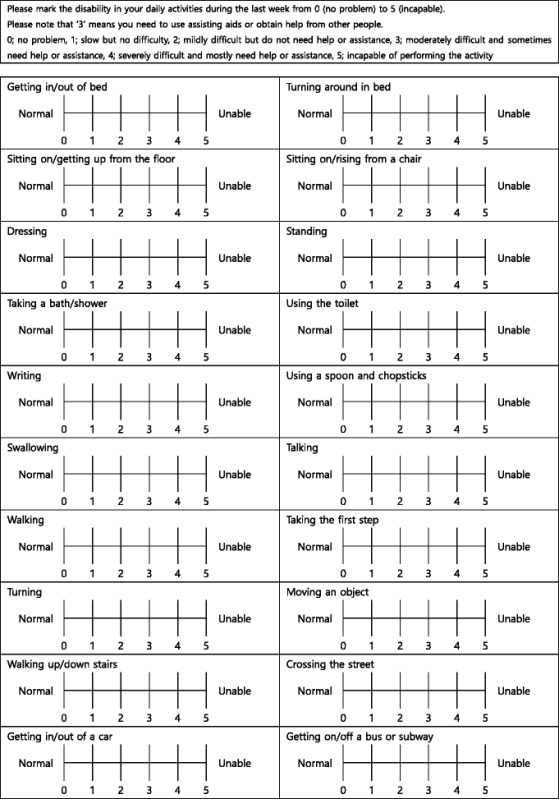
The new activities of daily living questionnaire

## Appendix

### Appendix 1

**Table 6:** Preliminary 45-item questionnaire

Please check the daily activities that you find difficult to do during last week. Please list the three activities that are most important to you.	
Household	Outdoor
Getting in/out of bed	Taking the first step
Turning around in bed	Walking outside
Sitting on the floor	Turning
Getting up from the floor	Stopping walking
Dressing	Walking up/down stairs
Sex life	Crossing the street
	Running
Sitting on and rising from a chair	Getting in/out of a car
Sitting upright	Getting on/off of a bus or subway
Standing	Driving a car
Walking inside	
Grasping and releasing an object	If you (or the patient) cannot walk
Moving an object	Moving from the bed or a chair to a wheelchair
Writing	Using a wheelchair
Wearing shoes	
	Social
Brushing teeth	Talking
Getting in/out of the bath	Using the phone
Taking a bath/shower	Shopping
Using the toilet	Going out
	Walking around the neighborhood
Preparing a meal	Working
Using a spoon and chopsticks	Taking exercise
Swallowing	Doing hobbies
Washing the dishes	Traveling
Cleaning the house	
Washing the clothes	Activities most important for you.
	1.
If you find any other activities difficult that are not in this list, please list them below.	2.
	3.

## Abbreviations

PDQL: Parkinson’s disease quality of life
ADL: Activities of daily living
HY: Hoehn and Yahr
K-MMSE: Korean version of mini-mental status examination
PD: Parkinson’s disease
SEADL: Schwab and England ADL scale
UPDRS: Unified Parkinson’s disease rating scale
